# Development and Evaluation of Evidence-Informed Clinical Nursing Protocols for Remote Assessment, Triage and Support of Cancer Treatment-Induced Symptoms

**DOI:** 10.1155/2013/171872

**Published:** 2013-02-18

**Authors:** Dawn Stacey, Gail Macartney, Meg Carley, Margaret B. Harrison, The Pan-Canadian Oncology Symptom Triage and Remote Support Group (COSTaRS)

**Affiliations:** ^1^University of Ottawa, 451 Smyth Road, Ottawa, ON, Canada K1H 8M5; ^2^Children's Hospital of Eastern Ontario, 401 Smyth Road, Ottawa, ON, Canada K1H 8L1; ^3^Queen's University, 78 Barrie Street, Kingston, ON, Canada K7L 3N6

## Abstract

The study objective was to develop and evaluate a template for evidence-informed symptom protocols for use by nurses over the telephone for the assessment, triage, and management of patients experiencing cancer treatment-related symptoms. Guided by the CAN-IMPLEMENT© methodology, symptom protocols were developed by, conducting a systematic review of the literature to identify clinical practice guidelines and systematic reviews, appraising their quality, reaching consensus on the protocol template, and evaluating the two symptom protocols for acceptability and usability. After excluding one guideline due to poor overall quality, the symptom protocols were developed using 12 clinical practice guidelines (8 for diarrhea and 4 for fever). AGREE Instrument (Appraisal of Guidelines for Research and Evaluation) rigour domain subscale ratings ranged from 8% to 86% (median 60.1 diarrhea; 40.5 fever). Included guidelines were used to inform the protocols along with the Edmonton Symptom Assessment System questionnaire to assess symptom severity. Acceptability and usability testing of the symptom populated template with 12 practicing oncology nurses revealed high readability (*n* = 12), just the right amount of information (*n* = 10), appropriate terms (*n* = 10), fit with clinical work flow (*n* = 8), and being self-evident for how to complete (*n* = 5). Five nurses made suggestions and 11 rated patient self-management strategies the highest for usefulness. This new template for symptom protocols can be populated with symptom-specific evidence that nurses can use when assessing, triaging, documenting, and guiding patients to manage their-cancer treatment-related symptoms.

## 1. Introduction

Adults undergoing cancer treatments often experience distress from treatment-related symptoms [1, 2]. Helping patients manage these symptoms can relieve some distress but more importantly better symptom management may lead to safer care for some symptoms that can progress to be life threatening [3]. Given that most chemotherapy and radiation therapy is provided through ambulatory programs, and patients experience treatment-related symptoms at home, telephone is the easiest way to access oncology health professionals [4, 5]. Thus, an important service for patients is telephone access to healthcare professionals for self-care guidance and triaging symptoms to the appropriate level of care. According to recent surveys, 88% of ambulatory oncology programs in Ontario reported that nurses respond to incoming calls from patients for symptom management and 54% of oncology nurses in Canada provide remote symptom support by telephone or email [6, 7]. 

Current research is limited to nurse initiated telephone calls to specific oncology patient populations as a follow-up posttreatment to address informational and psychosocial needs, or to monitor for recurrent disease. These types of outgoing telephone-based nursing services have been shown to be feasible and effective for meeting the information needs of patients with specific types of cancer [8–16]. Furthermore, telephone follow-up calls have been positively received and are deemed acceptable by patients and nurses [8, 9, 11, 17–19]. Telephone-based interventions reported in these studies are focused on cancer disease specific questions to ask in the follow-up calls without details on management of the oncology treatment related symptoms.

Key elements necessary for quality telephone-based nursing services that minimize risk of litigation include access to protocols to guide the assessment and advice provided, documentation of calls, quality assurance monitoring, and training [20, 21]. However, access to and the ways symptom protocols are used vary across oncology programs and nurses [6, 7]. Most programs in Canada that reported using protocols indicated they were using those available through Cancer Care Ontario since 2004. Often these protocols were not being integrated into clinical practice. Another potential source of synthesized evidence to guide symptom management is clinical practice guidelines [22, 23]. However, cancer symptom focused clinical practice guidelines are not formatted for use by telephone and the publicly available telephone symptom management protocols did not provide references to these sources of evidence [6, 7]. This highlights a gap between the evidence to support symptom management and the tools to facilitate use of evidence in clinical practice. Clinical practice protocols as knowledge translation tools can reduce the gap between scientific evidence and current practice by presenting the best available evidence and using a format that is sensitive to how nurses think and what nurses do [22, 24].

The overall aim of this study was to develop and evaluate a template for evidence-informed symptom protocols for use by nurses over the telephone for the assessment, triage, and management of patients experiencing cancer-treatment-related symptoms. For the purposes of this study, a protocol is defined as an agreed upon standardized approach to guide nursing practice. It is based on the best available scientific evidence and formatted for ease of use to fit with usual clinical routines or practices for a specific patient situation.

## 2. Materials and Methods

Methods were guided by the CAN-IMPLEMENT© methodology [25]. This framework was chosen because of its focus on adapting current guidelines. CAN-IMPLEMENT© is an adaptation and implementation planning resource designed to facilitate guideline adaptation and knowledge activation. The three-part resource (Guide, Library Science Supplement, and Toolkit) provides practical guidance for those interested in using already developed guidelines, adapting those recommendations for local use, and preparing for implementation. The procedures that guided our study were to (a) establish the research questions; (b) conduct a systematic review of the literature to identify clinical practice guidelines and systematic reviews for cancer-treatment-related symptom management; (c) appraise methodological quality of identified guidelines and systematic reviews; (d) iteratively develop a template for clinical nursing symptom specific protocols for use on the telephone with nurses and researchers; and (e) evaluate the protocol template for acceptability and usability. To provide an exemplar, we chose to focus on two different symptoms commonly associated with cancer treatments: diarrhea and neutropenia with fever. This project was a part of the larger Canadian Guideline Adaptation Study and ethics approval was received from the Queen's University Research Ethics Board, Kingston, Ontario, Canada (REB number NURS-211-07).

### 2.1. Research Questions to Inform Protocols

 To determine the evidence required to inform the development of clinical nursing protocols for the assessment, triage, and management of cancer patients experiencing treatment-related symptoms, we first identified five key questions (1) How are symptoms defined? (2) What criteria are used to assess the symptom? (3) How can a patient be risk stratified (e.g., high risk versus low risk of negative outcome) based on the symptom assessment findings? (4) How should patients' symptoms be managed (e.g., education in self-care and referral for consultation of other health professionals)? (5) What followup is required for ongoing symptom monitoring?

### 2.2. Search Strategy

The search strategy was designed in collaboration with a health sciences librarian (AR-W). Inclusion and exclusion criteria were defined using the PIPOH (Population, Intervention, Professionals/Patients, Outcomes, Health Care Setting) framework [26]. Based on a series of exploratory searches conducted in 2007 in Ovid interface to identify guidelines and systematic reviews in the databases Medline, Embase, CINAHL, and Psychinfo, the PIPOH criteria were refined (see Table 1). For example, searching for guidelines explicitly about telehealth practice was too limiting therefore the inclusion criteria were expanded to include any guideline about symptom management related to cancer care. The final search of each electronic database was conducted in July 2008 and was limited to the previous 5 years, from 2003 to 2008 (see Table 2). Using the key terms developed for the electronic searches, grey literature searches were conducted on websites known or suspected to have practice guidelines related to cancer care and known guideline clearinghouse websites. As citations were identified in the various searches, they were placed into a RefWorks database and duplicates were removed. To ensure currency of selected guidelines, the primary author of each guideline was contacted and asked the following: (a) has there been any more recent version and/or plans to update the guideline? and (b) are you aware of any new evidence that might affect the guideline recommendations? 

### 2.3. Screening

Citations were screened for eligibility by two independent reviewers using a three-stage process: titles only, title plus abstract, and full-text screening. Levels one and two were completed by GM and DS to judge the citation as include, exclude, or unsure. All citations rated as include or unsure by at least one reviewer were advanced to the next level. Full text documents were screened for eligibility (GM, DS) and discrepancies were resolved by consensus with the health sciences librarian. 

### 2.4. Appraisal of Methodological Quality

Each included guideline was appraised by four independent raters using the AGREE Instrument (Appraisal of Guidelines for Research and Evaluation) (DS, GM, MC, DC, KC, BS, DB, ML, AB). The AGREE Instrument has proven reliability and validity for assessing the quality of clinical practice guidelines [27]. AGREE ratings were entered into a spreadsheet. AGREE scores were calculated for each of the five AGREE domains and the overall recommendation, then standardized out of 100. AMSTAR was used to appraise the quality of the systematic reviews by two independent raters [28]. AMSTAR scores had a total possible score of 22 given that there were 11 questions and two appraisers. AMSTAR ratings were entered into a spreadsheet and scores tabulated.

### 2.5. Data Extraction

Following appraisal of identified guidelines and systematic reviews for quality, data was extracted independently by two team members and included: title, publication year, currency survey results, quality appraisal scores, overall recommendation, strengths, limitations, related algorithms/tools, and evidence to answer research questions with level of evidence. A recommendations matrix was populated with the extracted data to allow for comparison across citations [9].

### 2.6. Iterative Protocol Development

We convened a pan-Canadian panel of 24 stakeholders with a range of expertise in oncology nursing, knowledge translation, CAN-IMPLEMENT© methodology, library sciences, health services research, and electronic systems tools from eight Canadian provinces. Participants were involved in (a) reviewing findings from the systematic literature search, screen, and quality appraisal; (b) reaching consensus on symptom-specific recommendations to include in the protocol; (c) providing iterative feedback as the protocols were developed (via email and in face-to-face meetings). In pairs, participants engaged in role play with the protocols in order to further evaluate their usability and were asked to provide oral feedback during the meeting. A meeting evaluation feedback questionnaire given to participants consisted of eight multiple choice and two open end response type questions pertaining to evaluation of the meeting (i.e., objective met, information presented, and enough time) and evaluation of the protocols. Specifically, participants were asked whether the protocols provided an accurate reflection of the current scientific knowledge and whether they were willing to use them in practice. 

### 2.7. Methods to Evaluate the Symptom Protocol

Preliminary acceptability and usability testing of the protocols was conducted with oncology nurses from across Canada using a brief survey tool. The tool asked nurses to rate the protocol(s) based on their perceived ease of use, amount of information provided, use of appropriate terms, likelihood to fit with clinical work flow, helpfulness of the various elements provided (e.g., assessment criteria, severity rating and self-care strategies), and provided space for general comments. The questions were taken from previous surveys used for implementing knowledge tools in clinical practice [29, 30]. The survey consisted of eight questions including seven multiple choice and one likert scale plus three open ended responses for information to be added or removed, general comments, and suggestions for improvement. The survey was self-reported. Both quantitative and qualitative findings were entered into a spreadsheet and reported descriptively.

## 3. Results

### 3.1. Systematic Review

**Figure 1:**
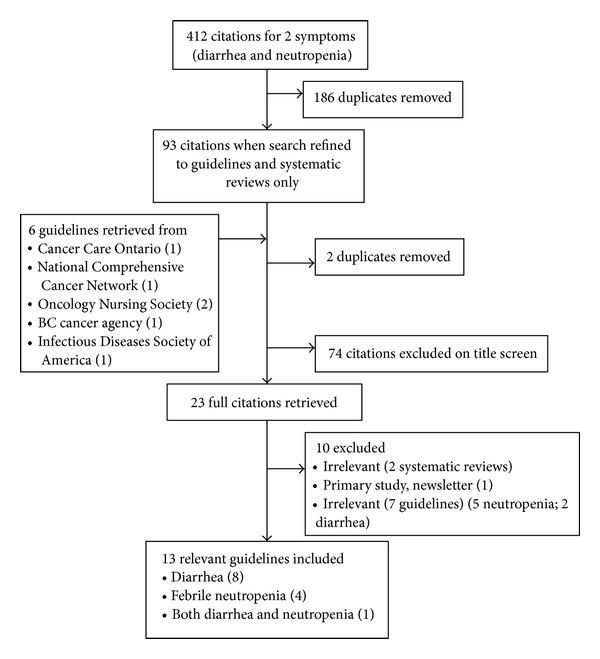
Flow of citations through screening process.

A total of 412 citations were identified for two cancer-treatment-related symptoms of diarrhea and fever with neutropenia (see Figure 1). After removing duplicates and narrowing the search parameters to guidelines and systematic reviews, we identified 93 citations. Grey literature searches identified six guidelines from cancer or cancer-related organizations. Of 93 citations, 74 were excluded based on title review, 10 were removed after full-text review (e.g., irrelevant, primary study, editorial), and 13 were included. Examples of why citations were identified as irrelevant include beyond the scope of nursing practice (i.e., prescribing medication), about prevention as opposed to assessment, triage and management, or did not pertain to the target population (i.e., endoscopy patients, chronic diarrhea). The 13 citations included 8 focused on diarrhea (6 guidelines and 2 systematic reviews), 4 febrile neutropenia (4 guidelines only), and 1 guideline including both symptoms. 

Diarrhea guidelines were found in peer-reviewed publications (*n* = 3) [31–33], British Columbia Cancer Agency (*n* = 2) [34, 35], the Oncology Nursing Society (*n* = 1) [36], and Cancer Care Ontario (*n* = 1) [37] (Table 3). Both systematic reviews of diarrhea management were from the Cochrane Library (selenium for alleviating treatment side effects) [38]; and Chinese medical herbs for chemotherapy side effects in colorectal cancer patients [39]. The febrile neutropenia guidelines were found in national organizations (*n* = 2) [40, 41], peer-reviewed publications (*n* = 2) [42, 43], and Cancer Care Ontario (*n* = 1) [37] (Table 4). 

In February 2009, the currency survey checking for updated evidence about diarrhea was completed by all the seven guideline developers and both systematic review authors. Only one guideline [32] had been updated and the new guideline was added [44]. The currency survey for fever with neutropenia was completed in February 2009 by one of the three guideline authors and no guidelines had been updated.

### 3.2. Quality of Evidence

**Figure 2:**
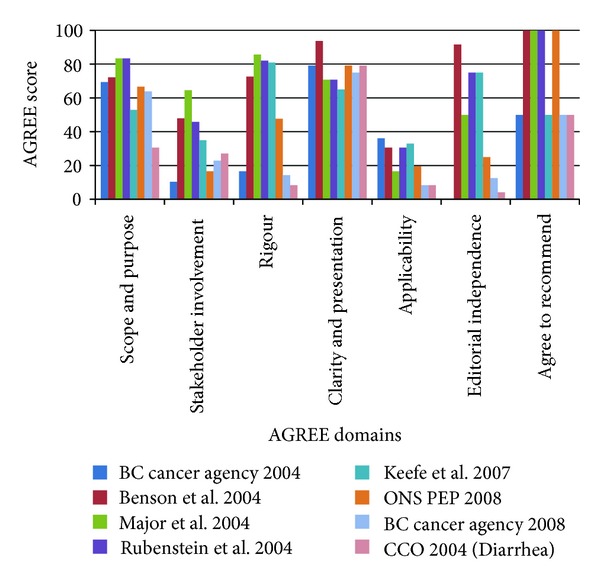
Diarrhea guideline AGREE scores for each of the AGREE domains with higher scores indicating higher quality guidelines (*n* = 8 guidelines).

For clinical practice guidelines relevant to diarrhea (*n* = 8), AGREE domains were rated higher for clarity of presentation (median 77; range 65 to 94), guideline purpose (68; 31 to 83), and rigour of development (60; 8 to 86) (see Figure 2). There were lower ratings for editorial independence (38; 4 to 92), stakeholder involvement (31; 10 to 65), and applicability (25; 8 to 36). Four of the guidelines which were recommended by all four raters to inform the development of the protocols also had the highest rigour scores. The remaining guidelines with lower rigour scores were each recommended by two of four raters. Raters indicated that these documents of lower methodological quality may still be useful for informing the presentation of the protocols as opposed to contribution of evidence. The two systematic reviews both scored highly in terms of methodological quality (AMSTAR ratings of 19/22 (86.4%)) but reviewers agreed neither should to be used to inform the development of the protocols given their content did not answer any of the research questions. 

**Figure 3:**
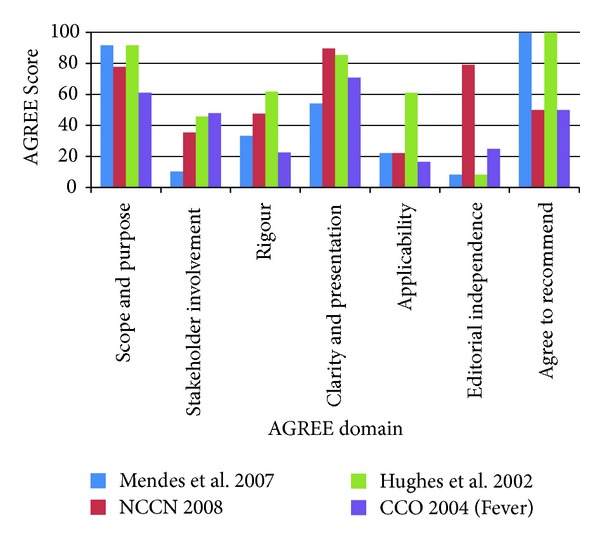
Febrile neutropenia guideline AGREE scores for each of the AGREE domains with higher scores indicating higher quality guidelines (*n* = 4 guidelines).

 For clinical practice guidelines relevant to fever with neutropenia (*n* = 5), one guideline was not recommended by any raters and was removed from the analysis. For the remaining clinical practice guidelines relevant to fever with neutropenia (*n* = 4), AGREE domains were rated higher for scope and purpose (median 84.7; range 61 to 92) and clarity of presentation (78.1; 54 to 90) (see Figure 3). There were lower ratings for stakeholder involvement (40.6; 10 to 48), rigour of development (40.5; 23 to 62), applicability (22.2; 17 to 61), and editorial independence (16.7; 8 to 79). All four raters recommended two of the guidelines while the other two guidelines were recommended by two of four raters.

### 3.3. Iterative Protocol Development

In March 2009, a group of 14 experts from the research team (AR-W, BS, CT, CK, DC, DS, EG, GM, KC, MH, MC, MS, RS, JV) and two local oncology nurses (MR, JR) convened in Ottawa, Ontario, and reached consensus on the recommendations for symptom-specific protocols based on the current evidence and the protocol format after a review of existing symptom protocols identified in oncology, palliative care, and primary care. The protocol template has five recommendations for the nurse to (a) assess symptom severity, (b) triage patient for symptom management based on highest severity; (c) review medications being used for the symptom, (d) review self-management strategies (presented using motivational interviewing techniques [45]), and (e) summarize and document the plan agreed upon with the patient. For each recommendation, there are questions and prompts for the nurse to explore the symptom experience with the patient (http://www.cano-acio.ca/triage-remote-protocols  example protocol). The resulting protocol was focused specifically on the symptom and could be combined with general assessment information collected on all patients. The meeting evaluation feedback questionnaire completed by 11 of 16 participants revealed that all participants thought the final symptom protocols would be an accurate reflection of the current scientific knowledge and for those whom it was relevant (*n* = 7) they would be willing to test the protocols in clinical practice. After trying the protocols in role play exercises, they were revised using plain language to facilitate communication between nurses using the protocol(s) and patients. It was apparent that the protocols required training in their use particularly given that few rated the protocols as self-evidence to use. A set of principles were finalized for creating the symptom protocol template (see Table 5).

### 3.4. Evaluation of the Symptom Protocol for Acceptability and Usablity

 Preliminary feedback to establish the protocol's acceptability and usability was obtained from 12 oncology nurses. Nurses rated the amount of information as just right (10 nurses), too much (1), or no answer (1). When asked if terms were appropriate, 10 agreed, 1 disagreed, and 1 gaveno answer. All 12 nurses indicated the font type, size, and icons made readability good. When asked if using the protocol would be self-evident, 5 agreed, 7 disagreed with areas requiring more guidance being the assessment (*n* = 6), documentation (*n* = 4), and triage (*n* = 2). Nurses were divided on rating the amount of space for entering data (6 enough, 4 not enough, and 2 no answer). When asked if the protocol would fit with clinical work flow, 8 agreed, 1 disagreed, and 3 did not answer the question. Nurses rated the patient self-management strategies the highest for usefulness of information followed by review of medications, triage of severity, and documenting. Suggestions to improve the protocols that were subsequently acted upon were explaining levels of evidence (changed level of evidence to type of evidence), interpreting assessment, adding more information (e.g., if symptom worsens in triage section; in self-care, discuss medications with physician or pharmacist), and using more plain language (e.g., provide medication trade and generic names; change decreased performance status to interfering with activities of daily living). Suggestions to improve the protocols that were not specifically acted upon were the need for protocols that incorporate more than one symptom and adding space to document emotional support. An open space was added for additional comments and could be used to document related emotional issues. General feedback included clear, user-friendly, comprehensive assessment, very thorough yet concise, offers direction without needing to seek more information, and excellent self-care strategies. Nurses liked the tick boxes that could save time from excessive charting, the ease of tool completion while talking, and clear differentiation between mild, moderate, and severe symptoms. Others appreciated the way the protocols linked the evidence for use in practice. Unresolved issues were the need to provide training on how to use the protocols in clinical practice, having electronic applications, and the potential for local adaptation given the different types of resources available across the various ambulatory oncology programs in Canada (e.g., hours of service and ability to link to physicians).

## 4. Discussion

 The CAN-IMPLEMENT© methodology [25] provided a solid framework and systematic approach for the development of a template for cancer-treatment-related symptom protocols for use by nurses over the telephone. This framework was chosen because of its focus on adapting current guidelines. As stated in CAN-IMPLEMENT©, one group may select one specific, relevant, and high quality guideline for adaptation to their local context; another may identify relevant segments of several high quality guidelines to customize the information to meet their needs. It was impossible to choose a single guideline and therefore our protocol was based on the latter. Interestingly, the systematic reviews were inadequate for informing the protocols due to their narrow scope. Factors which strengthened this project were the systematic and rigorous methods used to search and screen available guidelines, the ability to see consistencies across guidelines via a recommendations matrix, being able to use information from multiple guidelines starting with those with the highest quality appraisal ratings, a transparent and reproducible process for the evidence used to inform the protocol, and a thorough approach to document the symptom experience.

 Although clinical practice guidelines are described as knowledge translation tools [46], they are not readily useable in clinical nursing practice for oncology symptom management. In fact, they are often not integrated into clinical practice for any clinical setting or discipline. We were then challenged to determine a way for translating evidence into a new type of knowledge tool that was sensitive to how nurses think and what type of care they provide in clinical practice. While developing the new protocol template, our intention was to address the necessary elements to meet the criteria specified by the Rigour of Development domain of the AGREE Instrument. Specifically, this relates to being transparent about the process used to search, screen, and synthesize the evidence and the methods used to inform the recommendations [47]. The end result is a user friendly protocol that translates evidence from clinical practice guidelines for use in clinical practice.

Feedback from various stakeholders during the iterative development of the protocols clearly indicate, the need for training. Our findings are consistent with nursing professional practice teletriage guidelines that require training in teletriage [20, 21] and with findings from systematic reviews of effective interventions for implementing evidence into clinical practice [48]. A review of 81 randomized trials found that educational meetings improve patient care by 6.0% (range ±1.8% to 15.3%) with higher effects observed when health professionals attended the training and more interactive learning activities were used [48]. The only other intervention that showed higher improvements in patient care was having a local opinion leader (12% improvement; range 6.0 to 14.5%). One qualitative study of nurses' experiences providing telephone followup after treatment highlighted the need to further develop nurses' skills, including communication skills to be able to provide this type of intervention [17]. Therefore, training in how to use these new protocols will be important to ensure they are being used the way they were intended and to help users understand those sections that were identified as being less intuitive. 

 To develop the protocol template, we specifically chose to narrow the target audience to oncology nurses providing telephone-based services for patient initiated calls and avoided aiming to develop a protocol that would be relevant to all oncology service providers. However, the protocol is likely relevant for nurses providing face-to-face patient care as well, and in particular for nurses visiting patients in their homes. Further research is needed to evaluate their acceptability, usability, and relevance to nursing practice across different oncology settings (i.e., hospital, clinic, and home care), and not limiting to telephone-based services. Two main limitations were the variable methodological quality of included guidelines and the minimal amount of information that could be used from any one higher quality guideline.There were variable quality ratings for the included guidelines and including guidelines of lower quality may result in the protocols recommending less evidence-based actions. Although we included findings from lower quality guidelines, we did so to indicate consistency across guidelines, with a goal of being transparent about how the information was used (e.g., referencing guidelines within the protocol and providing their rigour ratings based on the AGREE assessment in the reference list). For example, if a specific recommendation was present in a lower quality guideline and higher quality guideline, both documents were cited in order to show consistency across the source documents. Finally, lower quality guidelines were considered useful primarily for formatting and presentation of information within the protocol. Another potential limitation was only including guidelines rather than searching for individual studies. The main reason for doing so was to use a synthesis of evidence that has the potential of increasing the stability of the findings [49]. About half of the included guidelines (those with higher rigour scores) were based on a synthesis of primary articles to inform their recommendations. Given these findings had already been synthesized into a quality guideline it would have been redundant to consult these primary studies to inform the development of the protocol. 

## 5. Conclusions

 Guided by the CAN-IMPLEMENT© methodology, we developed and evaluated a template for creating clinical nursing symptom protocols to use with patients experiencing symptoms while receiving cancer treatments. This new protocol template which puts evidence into an actionable format is a considerable advancement to the CAN-IMPLEMENT© for point-of-care use of evidence. New protocols created in this initiative provide an evidence-informed and user friendly approach for nurses to systematically assess, triage, document, and if appropriate guide patients in managing their symptoms at home. Furthermore, these types of clinical protocols are consistent with Accreditation Canada's requirements for enhancing safe practices within organizations [35]. However, training on how to use the protocols is important to ensure they are used properly and safely. The process of translating evidence from guidelines into practice-ready symptom protocols is relevant for other cancer symptoms and could be considered for other patient populations. Subsequent application and evaluation of the process by other groups will be important to advance greater uptake of evidence into practice. More specifically, there is a need to consider protocols that apply a symptom cluster approach inclusive of emotional symptoms that could facilitate symptom management beyond a single symptom approach. Finally, there is a need to evaluate their impact on nursing, patient, and organizational outcomes.

## Figures and Tables

**Table 1:** Criteria for searching and screening eligibility of potential citations.

Criteria	Eligibility	Ineligible
Population	Adults with cancer on chemotherapy, hormone therapy, and/or radiation therapy	Surgery alone
Intervention(s)	Any cancer-treatment-related symptom intervention to assess, rate severity, or manage	
Professionals targeted	Nurses and other health professionals working in oncology services	
Outcomes	Appropriate referrals for medical consultation, safe management of symptoms, patients guided in self-care	
Healthcare setting	Telephone or email to patients at home receiving services through ambulatory oncology program	
Methodology	Clinical practice guideline or systematic review	
Language	English or French	Other languages
Publication dates	2002 or later	Prior to 2002

**Table 2:** Final search strategy of the electronic databases.

Database	Limits	Strategy neutropenia	Strategy for diarrhea
Ovid MEDLINE(R) (1996 to July week 1 2008)	2003–2008 English/French humans	(1) exp. neoplasms/(811201) (2) Neutropenia/(6657) (3) Febrile neutropenia.ab,ti. (2107) (4) 1 and 2 or 3 (4892) (5) Limit 4 to humans and year = “2003–2008” and english or french and guideline or practice guideline (6)	(1) exp. neoplasms/(811201) (2) exp. diarrhea/(11983) (3) Diarrhea$.ab,ti. (18069) (4) Diarrhoea$.ab,ti. (7932) (5) 1 and 2 or 3 or 4 (4866) (6) Limit 4 to humans and year = “2003–2008” and english or french and guideline or practice guideline (5)

EMBASE (1996 to 2008 week 28)	2003–2008 English/French humans	(1) exp. neoplasm/(877949) (2) Febrile neutropenia/(6834) (3) Febrile neutropenia.ab,ti. (2188) (4) 1 and 2 or 3 (6474) (5) exp ∗ practice guideline/(9361) (6) 4 and 5 (14) (7) limit 6 to human and year = “2003–2008” and english or french (12)	(1) exp. neoplasm/(876520) (2) exp. diarrhea/(57553) (3) Diarrhea$.ab,ti. (17055) (4) Diarrhoea$.ab,ti. (7543) (5) 1 and 2 or 3 or 4 (18526) (6) exp ∗ practice guideline/(9335) (7) 5 and 6 (18) (8) Limit 7 to human and year = “2003–2008” and english or french (17)

CINAHL—cumulative index to nursing and allied health literature (1982 to July week 1 2008)	2003–2008 English/French	(1) exp. neoplasms/(89596) (2) Neutropenia/(816) (3) Febrile neutropenia.ab,ti. (206) (4) 1 and (2 or 3) (399) (5) Limit 4 to year = “2003–2008” and english or french and practice guidelines (7)	(1) exp. neoplasms/(89596) (2) Diarrhea/(2449) (3) Diarrhea$.ab,ti. (2165) (4) Diarrhoea$.ab,ti. (705) (5) 1 and 2 or 3 or 4 (475) (6) Limit 5 to year = “2003–2008” and english or french and practice guidelines (0)

PsycINFO (2000 to July week 1 2008)		(1) exp. neoplasms/(10572) (2) Febrile neutropenia.ab,ti. (4) (3) 1 and 2 (0)	(1) exp. neoplasms/(10572) (2) Diarrhea/(147) (3) Diarrhea$.mp. (674) (4) Diarrhoea$.mp. (137) (5) 2 or 3 or 4 (768) (6) 1 and 5 (34) (7) treatment guidelines/(1947) (8) 6 and 7 (0)

**Table 3:** Characteristics of guidelines about diarrhea (*n* = 7).

Author (Year)	Country	Defines symptom	Criteria to assess symptom	Risk stratification	Self-care to manage symptom	Followup for ongoing monitoring	Other recommendations in guideline	Rigour score (raters would recommend)
Major et al. (2004) [31]	Canada	—	Limited	NCI-CTC grade 0 to 4	OTC medications	Reassess within 24 hours	Use of prescribed medication	86 (4/4)
Rubenstein et al./Keefe et al. (2004/2007) [32, 44]	USA; Australia	—	Limited	NCI-CTC grade 0 to 4	OTC medications	—	Use of prescribed medication	82 (4/4)
Benson et al. (2004) [33]	USA	—	√	Uncomplicated versus complicated	Dietary and OTC medications	Reassess within 24 hours	Use of prescribed medications	73 (4/4)
ONS-PEP (2008) [36]	USA	√	√	Similar to complicated above	Dietary and OTC medications	—	Use of prescribed medications	48 (4/4)
BC Cancer Agency (2004) [34]	Canada	—	Limited	NCI-CTC grade 0 to 4	Dietary and OTC medications	Reassess within 24 hours	Use of prescribed medications	17 (2/4)
BC Cancer Agency (2008) [35]	Canada	√	√	Nonurgent, urgent, emergent	Dietary and OTC medications	Reassess within 24 hours	Use of prescribed medications	14 (2/4)
Cancer Care Ontario (2004) [37]	Canada	√	√	Nonurgent, urgent, emergent	Dietary	—	—	8 (2/4)

√: present in the guideline; —: none; OTC: over the counter; NCI-CTC: National Cancer Institute Common Terminology Criteria.

**Table 4:** Characteristics of guidelines about febrile neutropenia (*n* = 5).

Author (year)	Country	Defines symptom	Criteria to assess symptom	Risk stratification	Self-care to manage symptom	Followup for ongoing monitoring	Other recommendations in guideline	Rigour score (raters would recommend)
Hughes et al. (2002) [41]	USA	≥38.3°C or ≥38°C for 1 hour and <500 cell/mm^3^ or <1000 cell/mm^3^ and expected to drop	See scoring index on Table 4 of Hughes et al. (2002) [41]	Scoring index on Table 4 of the paper for MASCC risk index	—	—	Use of prescribed medications	62 (4/4)
NCCN (2008) [40]	USA	Refers to Hughes et al. [41]	Temperature, neutrophil count	MASCC risk index	—	—	Use of prescribed medications	48 (2/4)
Mendes et al. (2007) [43]	Brazil	Refers to Hughes et al. [41]	Temperature, neutrophil count, sepses	Infectious diseases working group, German Society of Haematology and Oncology	—	Reassess in 12 to 24 hours if neutropenia only	Use of prescribed medications	33 (4/4)
Cancer Care Ontario (2004) [37]	Canada	≥38°C	Many items (temperature, chills, other symptoms, etc.)	Nonurgent, urgent, emergent	Monitor temperature; minimize infection risk	Take temperature every 2–4 hours	—	23 (2/4)
Gaillet (2007) [42]	France	Excluded due to low quality score; no data extracted						14 (0/4)

—: none; NCCN: National Comprehensive Cancer Network; MASCC: Multinational Association for Supportive Care in Cancer.

**Table 5:** Principles for clinical nursing protocol template features.

(1) Evidence-based using evidence from appraised clinical practice guidelines	
(2) Template should meet the criteria for being a guideline (AGREE II-rigour)	
(i) Systematic methods used to search for evidence	
(ii) Clear criteria for selecting the evidence (e.g. quality appraised guidelines)	
(iii) Methods for formulating the recommendations are described	
(iv) Health-related benefits, side effects and risks have been considered	
(v) Explicit link between recommendations and the supporting evidence	
(vi) Reviewed by experts prior to publication	
(3) Usable in practice beyond resource on the shelf	
(4) Be able to be integrated into the electronic health record and clinical practice (e.g., uses Edmonton Symptom Assessment System question that is frequently used to screen for symptoms)	
(5) Plain language to enhance patients' health literacy	
(6) If assessment criteria and triage for severity is vague or absent from guidelines, use the National Cancer Institute Common Terminology Criteria for Adverse Events.	
(7) Ensure consistency across guidelines (e.g., if blood in vomit listed as severe in the diarrhea guidelines, then it should also be severe on the bleeding guideline)	
